# Traumatic Brain Injury and Peripheral Immune Suppression: Primer and Prospectus

**DOI:** 10.3389/fneur.2015.00235

**Published:** 2015-11-05

**Authors:** Jon Hazeldine, Janet M. Lord, Antonio Belli

**Affiliations:** ^1^NIHR Surgical Reconstruction and Microbiology Research Centre, Queen Elizabeth Hospital Birmingham, Birmingham, UK; ^2^Institute of Inflammation and Ageing, University of Birmingham, Birmingham, UK

**Keywords:** traumatic brain injury, immune system, immune suppression, infection

## Abstract

Nosocomial infections are a common occurrence in patients following traumatic brain injury (TBI) and are associated with an increased risk of mortality, longer length of hospital stay, and poor neurological outcome. Systemic immune suppression arising as a direct result of injury to the central nervous system (CNS) is considered to be primarily responsible for this increased incidence of infection, a view strengthened by recent studies that have reported novel changes in the composition and function of the innate and adaptive arms of the immune system post-TBI. However, our knowledge of the mechanisms that underlie TBI-induced immune suppression is equivocal at best. Here, after summarizing our current understanding of the impact of TBI on peripheral immunity and discussing CNS-mediated regulation of immune function, we propose roles for a series of novel mechanisms in driving the immune suppression that is observed post-TBI. These mechanisms, which have never been considered before in the context of TBI-induced immune paresis, include the CNS-driven emergence into the circulation of myeloid-derived suppressor cells and suppressive neutrophil subsets, and the release from injured tissue of nuclear and mitochondria-derived damage associated molecular patterns. Moreover, in an effort to further our understanding of the mechanisms that underlie TBI-induced changes in immunity, we pose throughout the review a series of questions, which if answered would address a number of key issues, such as establishing whether manipulating peripheral immune function has potential as a future therapeutic strategy by which to treat and/or prevent infections in the hospitalized TBI patient.

## Introduction

Defined as “an alteration in brain function, or other evidence of brain pathology, caused by an external force” (1), traumatic brain injury (TBI) represents a major public health issue and socioeconomic problem worldwide. An injury with multiple underlying etiologies, which include road traffic collisions, assaults, and falls, TBI was the primary diagnosis in 177,239 hospital admissions in England in 2013–2014 (2), while in the United States, an estimated 1.7 million people sustain a TBI annually, culminating in ~1.4 million emergency department visits, 275,000 hospitalizations and 52,000 fatalities (3).

The clinical impact of TBI is highlighted not only by its high mortality rate [TBI contributes to a third of all injury-related deaths in the United States (3)] but by the significant long-term health complications reported by those who survive their injuries. Personality changes, anxiety, depression, short attention span, difficulty in information processing, as well as impaired co-ordination and balance are commonly reported by patients with non-fatal TBI, all of which pose significant challenges to their independent living, social reintegration, and working life (4, 5). Moreover, in addition to the physiological and psychological impact on the individual and their families, the effects of TBI extend to society as a whole. Across Europe in 2010, the economic cost of TBI was an estimated €33 billion (6), while in the United States, where around 5.3 million people are living with a TBI-related disability (7), the economic cost of TBI exceeds $70 billion per year (8).

Hospital-acquired infections (HAIs) are a frequent occurrence in patients with severe TBI. As a result of colonization by such organisms as *Staphylococcus aureus* (*S. aureus*), *Pseudomonas aeruginosa*, and *Haemophilus influenza*, lower respiratory tract and surgical site infections are two of the most common non-neurological complications following severe TBI with an incidence rate of 24–72 and 17–25%, respectively (9–14). While it is currently unclear whether HAIs in TBI patients are associated with an increased risk of death (11, 15–19), numerous studies have demonstrated that HAIs result in significant patient morbidity. For instance, when compared to their non-infected counterparts, TBI patients with HAIs have significantly longer intensive care unit and hospital lengths of stay (11, 15, 17, 20), a greater degree of organ system dysfunction (11, 15), an increased duration of mechanical ventilation (11, 15, 20), and a worse neurological outcome at follow-up (14, 21). This detrimental effect of infection on neurological recovery has also been demonstrated in rodent trauma models, where in mild TBI, Venturi et al. (22) showed that the superimposition of sepsis resulted in a significant exacerbation of postinjury motor deficit and cognitive impairment as well as increased neuronal cell death in the hippocampus (22).

Multiple risk factors have been shown to be independently associated with the development of infections in hospitalized TBI patients. These include nasal carriage of *S. aureus* and barbiturate use (10), prolonged hospitalization, surgical intervention and cerebrospinal fluid leak (13), damage to the central nervous system (CNS), and the need for intubation and mechanical ventilation (12). Of these, it is injury to the CNS that is considered the main factor driving the increased susceptibility of TBI patients to HAIs. Mechanistically, this has been attributed to CNS injury disrupting the well-balanced bidirectional communication that exists between the CNS and the immune system, resulting in a state of systemic immune suppression (23). Indeed, *in vitro* and *in vivo* studies have shown significant changes occur in the number, phenotype, and function of cells belonging to both the innate and adaptive arms of the immune system post-TBI, a phenomenon termed CNS injury-induced immune deficiency syndrome (23).

In this article, we provide a comprehensive overview of the impact that TBI has on the circulating peripheral immune system and propose that alongside the well-described CNS-driven mechanisms of immune suppression, a series of novel immune-mediated mechanisms contribute to the altered immune function observed postbrain injury. Moreover, we highlight key areas of research that need to be tackled by future studies if we are to gain a greater understanding of the mechanisms underlying TBI-induced changes in immunity and establish whether manipulation of the immune system could become a potential therapeutic avenue by which to treat the hospitalized TBI patient.

## Current Understanding of the Impact of TBI on the Peripheral Immune System

Via a combination of historical observations (24–30) and results derived from recent experimental studies (31–38), it is evident that TBI has a significant impact on both the function and composition of the circulating immune cell pool. In this section, we provide a detailed description of the changes that have been reported to occur in both the innate and adaptive arms of the peripheral immune system following TBI, focusing predominantly on results obtained from human-based studies (**Table 1**). Given that very few of these studies have provided a mechanistic explanation for the changes they report, we make reference, where appropriate, to studies conducted in other fields of research whose results we believe offer plausible explanations for some aspects of immune dysfunction that have been reported post-TBI.

**Table 1:** **Summary of TBI-induced changes in peripheral innate and adaptive immunity**.

	Frequency/absolute numbers	Function	Reference
Neutrophils	Increased frequency and absolute number	Increased basal ROS generation	(39)
		Enhanced fMLP and PMA-induced ROS production in the acute phase of TBI	(34)
		Impaired ROS generation to *E. coli* stimulation at day 9 postinjury	(35)
		Reduced phagocytosis	(36)
Monocytes	Increased absolute number of total monocytes	Increased intracellular IL-10 expression	(30)
			(39)
	Increased absolute number of anti-inflammatory “M2” monocytes		(35)
			(37)
Natural killer cells	Decreased frequency and absolute number of CD3^−^56^+^ NK cells	Increased percentage of perforin-positive CD3^−^56^+^, CD56^DIM^ and CD56^BRIGHT^ NK cells at day 1 postinjury	(40)
	Decreased frequency of CD56^DIM^ NK cells	Decreased percentage of perforin-positive CD3^−^56^+^ and CD56^DIM^ NK cells at day 4 postinjury	(41)
	No change in CD56^BRIGHT^ NK cell frequency		(31)
			(32)
			(38)
T cells	Decreased percentage and absolute number of CD3^+^, CD4^+^, and CD8^+^ T cells	Decreased proliferation and cytokine production in response to PHA stimulation	(25, 27–29)
			(40)
		Reduced LAK cytotoxicity	(41)
		Impaired DTH response	(31)

*DTH, delayed-type hypersensitivity; *E. coli*, *Escherichia coli*; fMLP, formyl-methionine-leucine-phenylalanine; IL, interleukin; LAK, lymphokine-activated killer; PHA, phytohemagglutinin; PMA, phorbol 12-myristate 13-acetate; ROS, reactive oxygen species; TBI, traumatic brain injury*.

### Innate Immunity

#### Neutrophils

Armed with an array of microbicidal mechanisms, which include reactive oxygen species (ROS) generation and phagocytosis, neutrophils are the most abundant leukocyte in human circulation and the first immune cell to arrive at a site of pathogenic challenge. Following TBI, leukocytosis occurs, the result of profound neutrophilia. Both in the immediate aftermath of TBI as well as in the days following injury, studies have shown that relative to healthy control values, both the absolute number (34, 35, 39) and frequency (34, 39) of circulating neutrophils are significantly increased, with one study reporting a 4.5-fold elevation in neutrophil numbers as early as 3 h post-TBI (39). This immediate neutrophilia is thought to result from TBI-induced increases in serum catecholamines and glucocorticoids (33). A surge in catecholamines would trigger the entry of marginated neutrophils into the circulation, while a rise in glucocorticoid levels would increase the size of the peripheral neutrophil pool by stimulating their release from bone marrow stores and by extending their lifespan and preventing circulating neutrophils from returning to the bone marrow for clearance (33, 42). In terms of the neutrophilia that has been observed up to 48 h postinjury (39), Junger et al. (34) recently reported significantly fewer neutrophils isolated from TBI patients undergo spontaneous apoptosis during an overnight culture *in vitro* when compared to those from uninjured controls (34). Thus, a TBI-induced extension in neutrophil life-span may explain the elevated number of circulating neutrophils that have been reported in the days following injury.

In the immediate hours and days following mild, moderate, or severe TBI, neutrophils exhibit increased ROS generation, both in their resting state (35) as well as in response to stimulation with the bacterial peptide formyl-methionine-leucine-phenylalanine (fMLP) and the protein kinase C activator phorbol 12-myristate 13-acetate (PMA) (34). In a recent study, Liao et al. attributed the enhancement in basal ROS generation to a TBI-induced increase in gp91^phox^ (35). The heme binding subunit of the ROS generating enzyme nicotinamide adenine dinucleotide phosphate (NADPH) oxidase (43), Liao et al. found gp91^phox^ expression was significantly higher in leukocytes isolated from TBI patients within 24 h of their injury when compared to leukocytes from uninjured controls (35). Interestingly, besides healthy controls, neutrophils from TBI patients generated significantly more ROS at rest and expressed higher amounts of gp91^phox^ than neutrophils obtained from patients that had suffered general trauma with no CNS injury (35), suggesting that TBI initiates a more robust oxidative response than non-head trauma. While the mechanism(s) underlying this exaggerated response are currently unknown, the loss of regulatory feedback control on immune function as a result of direct CNS injury has been proposed as one potential explanation (35).

In contrast to the enhanced ROS generation that characterizes the initial response to TBI (34, 35), neutrophils exhibit impaired ROS generation in the days following injury (36, 40). In a study of hospitalized TBI patients with moderate or severe brain trauma, Marks et al. (36) found that neutrophil ROS production on day 9 postinjury was significantly lower than that of healthy age- and sex-matched controls. Given that peak incidences of infection in hospitalized TBI patients occur 5–11 days after injury (21, 36), and neutrophils are the first line of defense against rapidly dividing bacteria, fungi, and yeast; then impaired ROS generation may be one mechanism underlying the increased susceptibility of hospitalized TBI patients to infection. With this in mind, a comparison of ROS generation between neutrophils isolated from non-infected and infected TBI patients revealed a significantly greater percentage of ROS producing cells in the uninfected group on day 6 postinjury (36).

Following TBI, the ability of circulating neutrophils to phagocytose immunoglobulin and complement-coated *Escherichia coli* (*E. coli*) is significantly reduced (35, 36). This impairment, which has been observed in the hours (35), days (35, 36), and weeks (35) following injury relates to both the percentage of phagocytosing cells as well as their individual cellular activity (number of *E. coli* engulfed per cell). The reduction in phagocytosis that occurs post-TBI is significantly greater than that which occurs following general trauma with no CNS injury (35) and has been suggested to reflect a compensatory mechanism aimed at minimizing the deleterious effects of the afore-mentioned TBI-induced increase in neutrophil ROS generation (35). In the setting of critical illness, a reduction in neutrophil phagocytosis akin to that observed following TBI has been reported in patients with suspected ventilator-associated pneumonia (44), an impairment that has been attributed to activation of the complement system (44, 45). More specifically, it has been shown that by activating the delta isoform of phosphoinositide 3-kinase, the complement component C5a inhibits the Rho GTPase RhoA, which is the key mediator of the actin polymerization that is required for neutrophils to engulf complement-coated pathogens (45, 46). While it is currently unknown as to whether *in vivo* exposure to C5a mediates the reduced uptake of complement-coated *E. coli* by neutrophils post-TBI, this is a mechanism worth investigating given that complement activation is a feature of the inflammatory response to TBI (47, 48) and in a murine model of TBI, C5a was found to influence the functional behavior of circulating neutrophils (49).

#### Monocytes

Monocytes are a heterogeneous population of blood-borne leukocytes that comprise ~5–10% of the circulating immune cell pool and based on the differential surface expression of CD14 and the Fc receptor CD16 are categorized into one of three distinct subsets: classical (CD14^++^16^–^), non-classical (CD14^+^16^++^), or intermediate (CD14^++^16^+^) (50). Currently, there is a paucity of information on what effect TBI has on monocyte biology, with a small number of groups focusing upon changes in their number, surface phenotype, and cytokine production.

In contrast to murine-based studies, where significant reductions in monocyte number have been observed in the early hours and days following TBI (37), human studies have shown that TBI leads to a significant elevation in the absolute number of circulating monocytes (35, 39). Whether this increase in monocytes, whose numbers in one study were 2.7-fold higher relative to healthy controls within 24 h of injury (35), is the result of an increase in all monocytes or reflects the expansion of specific subsets which is currently unknown. However, changes that have been described in the surface phenotype of circulating monocytes post-TBI suggest that the latter is responsible. The selectin CD62L, which mediates initial tethering and rolling of marginated cells along the vessel wall, is expressed exclusively on CD14^++^16^–^ classical monocytes (51, 52), while the β_2_-integrin CD11b is present in greater amounts on the surface of both classical and intermediate monocyte subsets than on non-classical monocytes (52). Following TBI, analysis of the monocyte pool in its entirety has revealed significant increases in CD11b and CD62L surface density (39). Given the expression profiles of these two adhesion markers, this data suggests that it is expansion of both the classical and intermediate subsets that drives the significant elevation in monocyte number observed post-TBI (35, 39).

In addition to surface phenotype, monocytes can be categorized into distinct subsets based upon their inflammatory properties and maturation profiles. In mice, Ly6C^+^ classical monocytes secrete proinflammatory cytokines and following entry into tissue mature into inflammatory M1 macrophages, while Ly6C^–^ non-classical monocytes secrete the anti-inflammatory cytokine IL-10 and differentiate into anti-inflammatory M2 macrophages (53). In a murine model of closed head injury, Schwulst et al. (37) found a significantly greater number of anti-inflammatory Ly6C^−^ monocytes in the peripheral circulation of TBI mice at 60 days postinjury when compared to sham controls, suggesting that TBI results in a shift toward an anti-inflammatory monocyte pool (37). In line with this observation, intracellular expression of IL-10 has been detected in monocytes isolated from human TBI patients immediately after injury (30), suggesting that akin to the situation described in mice, TBI in humans elicits an anti-inflammatory response in circulating monocytes.

#### Natural Killer Cells

Phenotypically defined as CD3^−^56^+^, natural killer (NK) cells are large granular lymphocytes renowned for their role in the recognition and elimination of virally infected, malignant, and transformed cells. In the days (31, 38) and weeks (38) following mild (38), moderate (38), and severe (31, 38) TBI, significant reductions have been reported in the absolute number and frequency of circulating NK cells. These measures correlated positively with both the Glasgow coma scale (GCS) score, suggesting that the changes observed could be a direct consequence of the brain injury (31, 38), and the Glasgow outcome scale (GOS), suggesting a relationship between immune status and physiological recovery (38). Based on the differential surface expression of CD56, NK cells can be categorized into two major subsets, namely cytotoxic CD56^DIM^ NK cells and immunoregulatory CD56^BRIGHT^ NK cells. Following TBI, the frequency of CD56^DIM^ but not CD56^BRIGHT^ NK cells is significantly diminished (31), indicating that it is a specific reduction in the CD56^DIM^ subset that is responsible for the above-mentioned decline in the total (CD3^−^56^+^) NK cell pool.

While it is currently unclear as to why TBI results in a significant decline in the number of circulating NK cells, one school of thought is that it represents a mechanism by which to prevent autoimmunity and CNS inflammation (38). *In vitro*, NK cells have been shown to kill resting microglia (54), the resident macrophage-like cell of the CNS that upon activation produces proinflammatory cytokines and acquires the ability to process and present antigens to CNS infiltrating T cells (55). Furthermore, it has been shown *in vivo* that CNS residing NK cells suppress the microglia-mediated induction of proinflammatory T helper 17 cells (56, 57). Thus, it has been proposed that the reduction in circulating NK cell numbers post-TBI is the result of NK cells entering the brain via the damaged blood–brain barrier, where through influencing microglial number and function they prevent the amplification of inflammatory processes and thus bystander damage in the CNS (38). An alternative mechanism that could explain the decline in circulating NK cell numbers post-TBI is cell death. As reported for other lymphocytes, glucocorticoid treatment *in vitro* has been shown to induce apoptosis in resting NK cells (58, 59). Thus, it is conceivable that the increase in serum glucocorticoids that accompanies TBI could trigger the induction of programed cell death in circulating NK cells, culminating in a significantly reduced peripheral pool.

NK cells eliminate transformed cells primarily via the granule exocytosis pathway, a contact-dependent mechanism of defense that involves the directed secretion of the pore-forming protein perforin onto the target cell surface. Within 24 h of severe TBI, significant increases have been reported in the percentage of perforin-positive CD3^−^CD56^+^, CD56^DIM^ and CD56^BRIGHT^ NK cells (32). As perforin expression is enhanced by proinflammatory cytokines (60), this increase in perforin positive NK cells has been proposed to be due to the leakage of interleukin (IL)-1, IL-6, and tumor necrosis factor-alpha (TNF-α) from the CNS into the circulation (32). Interestingly, by day 4 post-TBI, a time-point where the frequency of perforin-positive CD3^−^CD56^+^ NK cells correlates positively with GCS score, the percentage of perforin-positive CD3^−^CD56^+^ and CD56^DIM^ NK cells is significantly reduced relative to healthy controls (32), a decline that may contribute to the increased susceptibility of the hospitalized TBI patient to infection.

### Adaptive Immunity

Until recently, TBI was thought to have no impact upon B-cell biology, a belief based upon a handful of studies that had shown no alterations in the frequency, absolute number, or proliferative capacity of B cells following severe TBI (24, 28, 31, 40, 61). However, in two recent studies, autoantibodies specific for CNS proteins were detected in the serum of TBI patients, suggesting that following TBI, self-tolerance is broken and B cells initiate an immune response against brain-derived antigens. In the setting of mild TBI, Marchi et al. found that serum levels of the astrocytic protein S100B were higher in subjects that had experienced frequent sub-concussive head hits (SHH) and that the presence of this protein was accompanied by an elevation in anti-S100B autoantibodies (62). As raised S100B autoantibody levels were associated with poor performance in cognitive tests, and these autoantibodies reacted strongly toward both glial and neuronal cell epitopes, it was hypothesized that the generation of autoantibodies against CNS-residing proteins may represent a risk factor for premature neurodegeneration in individuals who suffer repeated SHH (62). In line with these observations, Zhang et al. detected autoantibodies against the astrocyte-residing intermediate filament protein glial fibrillary acidic protein (GFAP) and its breakdown products in serum samples obtained from 53 patients following severe TBI (63). These GFAP autoantibodies, which were detected as early as 4 days postinjury and whose levels positively correlated with GCS scores, were shown to induce glial cell injury *in vitro*, suggesting a potential pathophysiological role for B cells during the recovery phase of TBI (63).

Severe TBI results in a significant decrease in the percentage and absolute number of circulating T lymphocytes (31, 40, 41). This decrease, which has been observed within 24 h of injury (31, 41) as well as on day 4 postinjury (31), is the result of a significant reduction in both CD4^+^ T helper cells and CD8^+^ cytotoxic T cells (31, 40, 41). Currently, the mechanism(s) underlying this TBI-induced shrinkage of the circulating T-cell pool is unclear although results of a recent murine-based study offer a potential explanation. In a series of *in vivo* experiments, Nakai et al. (64) found that administration of B_2_-adrenergic receptor (B_2_AR) agonists resulted in a rapid reduction in the numbers of blood CD4^+^ and CD8^+^ T cells, with further investigations revealing this lymphopenia was the result of B_2_AR stimulation inhibiting lymphocyte egress from lymph nodes (64). Given that TBI results in increased circulating levels of catecholamines (65, 66), then lymphocyte retention in lymph nodes may be one mechanism by which to explain the significant reduction in circulating T cells that has been observed following TBI.

Alongside numerical changes, a multitude of functional defects have been reported in circulating T cells following TBI. While *in vitro* studies have shown that T cells isolated from patients with severe TBI exhibit reduced proliferation and cytokine production following phytohemagglutinin stimulation (26–28) as well as impaired lymphokine-activated killer (LAK) cytotoxicity following incubation with IL-2 (26, 27, 29), *in vivo* studies have revealed severe TBI results in suppressed delayed-type hypersensitivity (DTH) skin test responses to a range of antigens, which include Candida, mumps, and trichophyton (25, 28, 61). Mechanistically, this reduction in cell-mediated immunity has been suggested to be due to serum factors with immune-suppressive activity (29, 61) and the presence of suppressor lymphocytes that actively inhibit effector lymphocyte function (29).

## Neural Regulation of the Immune Response

Critical for the regulation of peripheral inflammatory responses is the bidirectional communication that exists between the immune system and the CNS. The brain senses peripheral inflammation via two main pathways; a neural pathway, which involves activation of the afferent sensory fibers of the vagus nerve, and a humoral pathway, in which circulating cytokines signal to the brain either by crossing the blood–brain barrier or by binding their cognate receptors on the brain microvasculature (67, 68). Interestingly, a functional lymphatic system within the CNS has recently been discovered, suggesting an additional level of communication between the CNS and the immune system (69, 70). Of particular interest with regards to TBI, Louveau showed B cells, T cells, and dendritic cells reside in meningeal lymphatic vessels and that these vessels serve as the primary route for the drainage of cerebrospinal fluid (CSF)-derived soluble and cellular constituents into cervical lymph nodes (CLNs) (70). Given that the contents of CSF have been shown to trigger immune responses in CLNs (71), this CNS lymphatic system may represent a novel means by which the immune system could detect alterations in brain physiology post-TBI.

Having sensed peripheral inflammation, the brain, in an effort to restore immune homeostasis, responds by signaling to the immune system via three immunomodulatory pathways: (1) the hypothalamic–pituitary–adrenal (HPA) axis (2) the sympathetic nervous system (SNS), and (3) the parasympathetic nervous system (PNS). In the context of TBI and peripheral immune suppression, the direct injury to the brain and/or the resulting ischemia, raised intracranial pressure and cerebral cytokine production that develops in the days and weeks postinjury is thought to disrupt the well-balanced interplay between the immune system and the CNS. Activation of the above-mentioned pathways of neural immune modulation in the absence of a peripheral inflammatory response culminates in a state of systemic immune depression that is thought to increase the risk of the TBI patient to nosocomial infection. Here, we provide a brief overview of the three above-mentioned neural pathways of immune modulation, highlighting key studies whose results suggest that these pathways may play a prominent role in mediating TBI-induced immune suppression.

### The Hypothalamic–Pituitary–Adrenal Axis

The HPA axis represents the primary hormonal pathway through which the CNS regulates peripheral immune function. Glucocorticoids, the main effector hormone of the HPA axis, modulate immune cell functions via binding to the glucocorticoid receptor, a cytosol residing ligand-dependent transcription factor, which modulates the expression of genes involved in cytokine production and such immune responses as cell trafficking and phagocytosis (72).

Via a series of mechanisms, which range from the destabilization of mRNA to inhibiting the binding of transcription factors to DNA (73), glucocorticoids suppress the production of several proinflammatory cytokines and inflammatory mediators, which include IL-1β, IL-6, IL-12, TNF-α, IFN-γ, nitric oxide, and prostaglandins (74–76). In contrast, glucocorticoids promote the secretion of the anti-inflammatory cytokines IL-4 and IL-10 (75). Through this modulation of inflammatory cytokine production, glucocorticoids effect immune cell trafficking by suppressing TNF-α-mediated increases in endothelial cell surface adhesion molecules (77) and influence T-cell biology by driving the differentiation of naive T lymphocytes to T helper 2 (T_H_2) cells and blocking T_H_1 development (78, 79).

In terms of their effects on immune cell function, glucocorticoids have been found to increase the T-cell-suppressive capacity of anti-inflammatory “M2” macrophages (80), suppress the cytotoxicity activity of NK cells by decreasing expression of the pore-forming protein perforin (81), inhibit neutrophil phagocytosis (82), induce lymphocyte apoptosis (83), and influence the antigen-presenting capacity of monocytes and dendritic cells by downregulating their expression of MHC class II and the costimulatory molecule CD86 (84, 85).

### The Sympathetic Nervous System

Through extensive innervation of primary (bone marrow and thymus) and secondary (lymph nodes and spleen) lymphoid organs and the expression of α and β adrenergic receptors on the surface of almost all circulating leukocytes, the SNS elicits a strong regulatory influence on peripheral immune function (86). In a series of *in vitro* experiments, catecholamines, the effector neurotransmitter of the SNS, or their pharmacological analogs have been shown to suppress, in a β-adrenergic receptor-dependent manner, a range of immune functions, which include neutrophil ROS generation, inflammatory cytokine production, and NK-cell cytotoxicity (86–91). With respect to cytokine production, pretreatment of whole blood or isolated monocytes, macrophages, or dendritic cells with epinephrine, norepinephrine, or the β_2_-agonist salbutamol has been found to significantly inhibit lipopolysaccharide (LPS)-induced IL-12 and TNF-α secretion (88, 89, 91, 92), a suppression that in the case of IL-12 was associated with an increase in intracellular cyclic adenosine monophosphate levels (92). While inhibiting proinflammatory cytokine production, catecholamines enhance the generation of anti-inflammatory cytokines, with epinephrine and norepinephrine treatment of whole blood or isolated monocytes significantly increasing both basal and LPS-induced secretion of IL-10 (88, 89, 93). Via these effects on inflammatory cytokine production, β-adrenergic receptor stimulation has been shown to indirectly influence the differentiation of naive T cells, promoting the development of T_H_2 cells and inhibiting the generation of T_H_1 cells (92). Given that TBI results in elevated circulating levels of catecholamines (65, 66), this catecholamine-induced shift in the T_H_1/T_H_2 balance in favor of anti-inflammatory T_H_2 cells may be one factor behind the increased susceptibility of TBI patients to infection.

In line with the *in vitro* effects of catecholamines, human- and murine-based *ex vivo* studies have demonstrated in a number of experimental settings an immune suppressive role for the SNS (90, 93–95), which in the case of direct CNS injury has been linked to the development of infection. In a murine model of stroke, Prass et al. found that inhibition of the SNS immediately following the onset of cerebral ischemia improved survival rates by preventing the spontaneous development of systemic bacterial infections that was observed in control mice (95). Underlying this protective effect was an enhanced immune response with *ex vivo* experiments, revealing that inhibition of the SNS prevented a stroke-induced reduction in IFN-γ production by circulating T lymphocytes; a function that the group found was crucial in controlling bacterial infections’ poststroke (95).

Both prospective and retrospective cohort studies have shown that the use of β-adrenergic receptor antagonists in adult TBI patients is independently associated with improved survival (96–98). Given that a reduced risk of ischemia, enhanced brain tissue metabolism and oxygen consumption as well as protection of the cardiovascular system are all potential benefits of this treatment protocol (98–101), it is likely that alterations to a number of physiological processes underlie this protective effect of β-adrenergic receptor blockade on patient survival post-TBI. However, *could reducing the incidence of infection by preventing SNS-induced immune suppression be an additional underlying mechanism?* This hypothesis has been rebuffed by some groups who reported a higher incidence of infectious complications among TBI patients treated with β-adrenergic receptor antagonists (97, 102). However, in these studies, patients who underwent this treatment protocol were older, more-severely injured and received a higher number of blood transfusions than their untreated counterparts (97, 102). Moreover, in one study, many of the patients did not commence their therapy until after the diagnosis of infection (97). Thus, to answer this question correctly, a study that performs β-adrenergic receptor blockade in a randomized, double-blinded, placebo-controlled trial of appropriately matched TBI patients that measures immune function, infection incidence, and outcome is required. Results of such a study would go a long way to addressing whether in the clinical setting of TBI, the surge in circulating catecholamines is causally linked to the subsequent development of immune suppression, and if so what impact this has on patient outcome.

### The Parasympathetic Nervous System

In a landmark study in 2000, Borovikova et al. described for the first time a vagus nerve-mediated parasympathetic pathway of immune modulation. After showing *in vitro* that treating primary human macrophage cultures with acetylcholine (ACh), the principal parasympathetic neurotransmitter significantly inhibited LPS-induced secretion of TNF-α, IL1-β, IL-6, and IL-18 without affecting the production of the anti-inflammatory cytokine IL-10, the group demonstrated *in vivo* a role for parasympathetic signaling in regulating systemic inflammatory responses (103). In a rat model of lethal endotoxemia, electrical stimulation of the efferent vagus nerve (which increases ACh release) significantly reduced serum TNF-α levels, whereas in mice subjected to vagotomy without electrical stimulation, TNF-α levels were significantly increased when compared to sham-operated controls (103). These two novel findings were the first to directly implicate efferent vagus nerve signaling in the regulation of proinflammatory cytokine production *in vivo*. Termed the cholinergic “anti-inflammatory” pathway, this neural mechanism of immune modulation is rapid, discrete, and localized when compared to the diffusible anti-inflammatory network (e.g., glucocorticoids and anti-inflammatory cytokines), therefore allowing for “real time” regulation of immune responses (103, 104).

As well as exerting anti-inflammatory effects in models of systemic inflammation such as severe sepsis (105, 106), haemorrhagic shock (107), and ischemia/reperfusion (108), a role for parasympathetic signaling in regulating localized peripheral inflammatory responses has been reported. In an experimental model of carrageenan-induced paw edema, electrical stimulation of the vagal nerve or local administration of ACh significantly attenuated the development of acute inflammation (109), an outcome that may be explained by the ability of ACh to inhibit endothelial cell activation and leukocyte recruitment (110).

The essential signaling component that links ACh release from efferent vagal fibers to peripheral immune suppression is the α7 subunit of the nicotinic ACh receptor (106, 111). *In vitro* treatment of human macrophages with antisense oligonucleotides specific for the α7 subunit has been shown to restore TNF-α secretion upon nicotine stimulation (111), while peritoneal macrophages isolated from α7 subunit deficient mice were found to be refractory to cholinergic agonists, secreting significantly greater amounts of TNF-α following LPS stimulation in the presence of ACh or nicotine when compared to macrophages from control mice (111). Moreover, *in vivo* studies have reported elevated serum levels of TNF-α, IL1-β, and IL-6 in α7-subunit-deficient mice after endotoxin challenge and shown that electrical stimulation of the vagus nerve in these mice fails to reduce this proinflammatory response (111).

Based on the results of the afore-mentioned studies and clinical data suggesting TBI results in enhanced parasympathetic activity (112), it has been hypothesized that overactivity of the vagus nerve may be responsible, at least in part, for the peripheral immune suppression that is observed following TBI (113, 114). An increase in vagal tone would occur as either a direct consequence of the TBI or as a result of elevated intracranial pressure, leading to systemic immune suppression that increases the susceptibility of the hospitalized TBI patient to infection (113, 114). Although plausible, it must be noted that no clinical studies have been performed to date that have investigated a role for parasympathetic signaling in mediating the increased incidence of infection in TBI patients. That said, if this theory is proven to be correct then modulating the activity of the vagus nerve could be a novel therapeutic approach by which to reduce not only the direct mortality attributable to infection but also the long-term effects of infection in respect to poor neurological outcome in TBI patients (113, 114).

## New Concepts in TBI-Induced Immune Suppression

**Figure 1:**
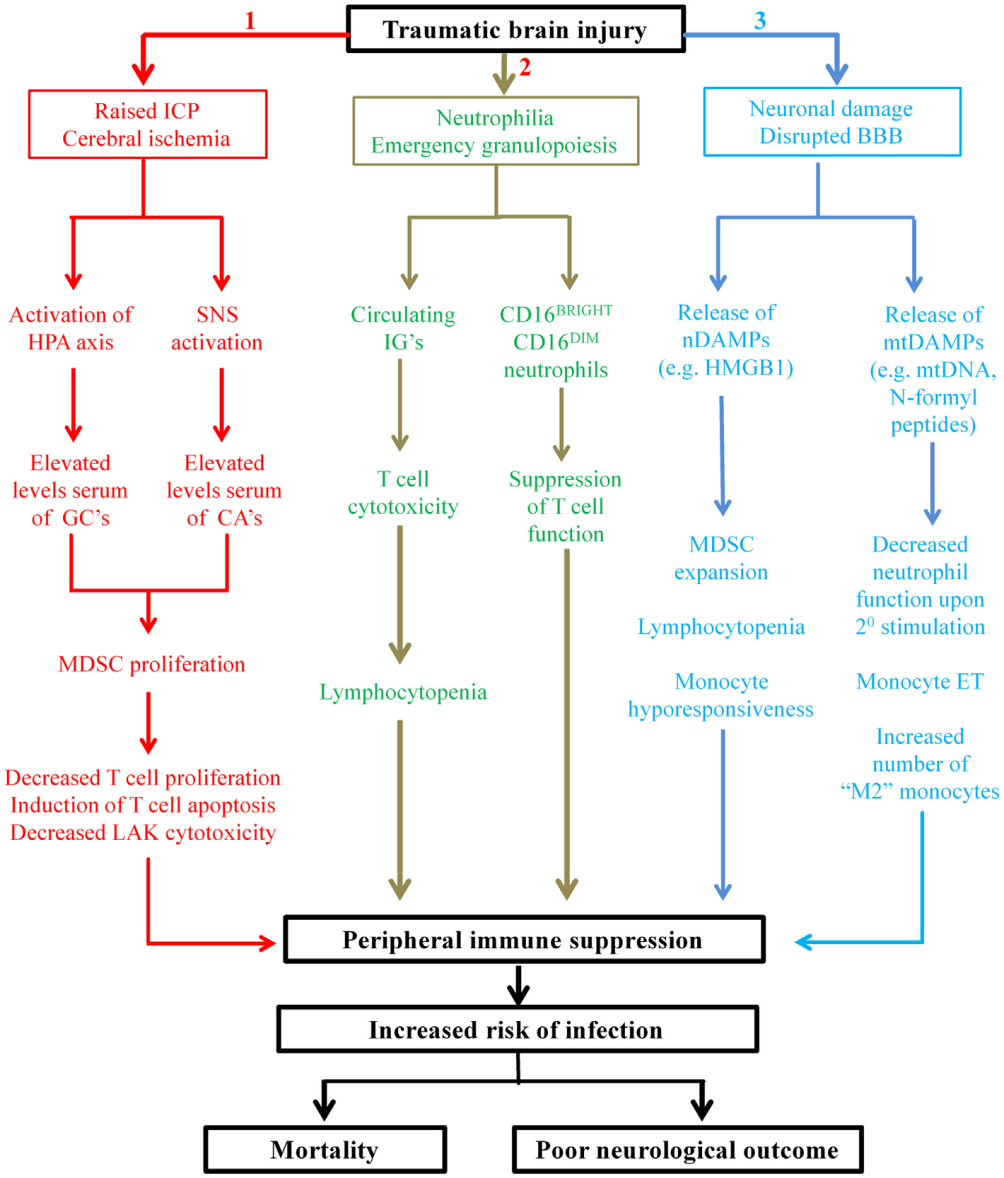
**Proposed mechanisms underlying TBI-induced changes in peripheral innate and adaptive immunities**. Applying the findings of studies performed on patients with general trauma (111, 113–115, 118, 122, 127) and CNS injury (131) to the hospitalized TBI patient, we propose three novel mechanisms contribute in part to the peripheral immune suppression that is observed post-TBI. Mechanism 1: secondary complications arising from TBI such as raised intracranial pressure (ICP) and cerebral ischemia result in elevated serum levels of glucocorticoids (GCs) and catecholamines (CAs) through activation of the HPA axis and SNS, respectively. GCs and CAs drive the proliferation and accumulation of myeloid derived suppressor cells (MDSC) in the circulation that subsequently inhibit T-cell function via depletion of arginine from the local environment and the production of reactive oxygen species (ROS). Mechanism 2: TBI triggers emergency granulopoiesis, which results in the emergence into the circulation of immature granulocytes (IGs) and the generation of a heterogeneous pool of neutrophils that contains CD16^BRIGHT^ CD16^DIM^-suppressive neutrophils. Through ROS generation, CD16^BRIGHT^ CD16^DIM^ neutrophils would suppress T-cell proliferation, while via direct cytotoxicity IGs would induce T-cell apoptosis and thus lymphocytopenia. Mechanism 3: neuronal cell death combined with disruption of the blood–brain barrier (BBB) would lead to the emergence into the circulation of both nuclear- and mitochondrial-derived damage-associated molecular patterns (DAMPs). The nuclear-derived DAMP high mobility group box 1 (HMGB1) would promote the expansion of MDSCs, monocyte hyporesponsiveness, and lymphocytopenia, while the presence of mtDAMPs would induce a state of systemic immune cell tolerance. More specifically, as a consequence of exposure to N-formyl peptides, circulating neutrophils would exhibit reduced antimicrobial activity upon secondary stimulation, while mitochondrial DNA (mtDNA) would promote a state of endotoxin tolerance in circulating monocytes. The consequence of all three of these proposed mechanisms is profound suppression of the peripheral immune system, resulting in an increased susceptibility to infection and an increased risk of poor outcome.

In spite of the recent increase in the number of studies that have investigated the effect of TBI on peripheral immunity (31, 32, 34–39), only one has provided a mechanistic explanation for their findings (35), with the majority merely reporting functional impairments relative to healthy controls (31, 32, 36, 37). This paucity of information on the mechanisms that underlie TBI-induced changes in immunity represents a major barrier that needs to be overcome if novel immune-based therapeutic strategies aimed at reducing the incidence of nosocomial infection in hospitalized TBI patients are to be developed. While disruption of the well-balanced interplay that exists between the nervous system and the immune system clearly plays a key role in TBI-induced immune suppression (23), it is likely that additional mechanisms are involved. In other forms of traumatic injury, non-neurological mechanisms of immune suppression have recently been described, with *in vitro*, *in vivo*, and *ex vivo* data revealing trauma results in the emergence into the circulation of suppressive immune cell subsets and damage-associated molecular patterns (DAMPs), with exposure to the latter inducing tolerance in peripheral immune cells (115–119). In this section, we discuss these recent studies, relate their findings to TBI and propose that that the presence of suppressive immune cells and DAMPs in the circulation contributes to the immune suppression that is observed following TBI (Figure 1).

### Myeloid-Derived Suppressor Cells as Mediators of *In Vitro* and *In Vivo* T-Cell Dysfunction Post-TBI

Myeloid-derived suppressor cells (MDSCs) are a heterogeneous population of cells consisting of myeloid progenitors and precursors of granulocytes, macrophages, and dendritic cells. First described in the field of cancer research and characterized since in a number of pathological conditions such as sepsis, autoimmunity, and trauma, MDSCs are potent suppressors of T-cell function (120, 121). Via the production of ROS and reactive nitrogen species, expression of arginase 1, and secretion of immunosuppressive cytokines, MDSCs inhibit T-cell proliferation, promote T-cell apoptosis, block translation of the T-cell CD3ζ chain, and mediate expansion of CD4^+^CD25^+^FoxP3^+^ regulatory T cells (120, 121).

In murine models of trauma (122) and chronic physiological stress (123), endogenous GCs and catecholamines have recently been shown to promote the expansion and accumulation of MDSCs. Thus, if we assume that akin to other forms of traumatic injury (115, 116), the initial act of brain injury is sufficient to cause MDSC release from the bone marrow then the increased serum levels of GCs and catecholamines found in TBI patients is an environment that favors MDSC proliferation and expansion. Consequently, it is not inconceivable that the presence of MDSCs in circulation could be one mechanism underlying the profound *in vivo* and *in vitro* T-cell suppression that occurs following TBI (25–28, 61). In fact, this scenario may have already been described. In a study published prior to the discovery of MDSCs, Quattrocchi et al. (29) found that when cocultured with lymphocytes isolated from patients with severe TBI, peripheral blood mononuclear cells (PBMCs) from healthy controls exhibited significantly reduced LAK cytotoxicity, suggesting the presence of suppressor lymphocytes in TBI patients (29). Interestingly, when performing standard PBMC isolation protocols using Ficoll-Hypaque density gradient centrifugation, MDSCs due to their reduced buoyant density reside in the same layer as lymphocytes (124). Thus, it may be that the lymphocytes isolated from TBI patients by Quattrocchi et al. (29) were contaminated with MDSCs and that it was these cells that were responsible for the reduction in T-cell cytotoxicity they reported.

Recently, MDSCs have been touted as a therapeutic target in the treatment of cancer with strategies aimed at either (1) deactivating MDSCs, (2) blocking their development, or (3) driving their differentiation into mature cells all currently under clinical investigation (125). If through future research, MDSCs are found to play a role in TBI-induced immune dysfunction then similar approaches may be worth considering as a potential therapeutic avenue for the treatment of nosocomial infections in the hospitalized TBI patient.

### Suppressive CD16^BRIGHT^ CD62L^DIM^ Neutrophils and CD16^DIM^ Immature Granulocytes as Potential Mediators of *In Vivo* T-Cell Dysfunction Post-TBI

Historically, neutrophils have been considered a relatively homogeneous cell population. However, in recent years it has become apparent that in times of severe systemic inflammation, distinct neutrophil subsets emerge into the circulation. Based on the differential surface expression of the Fc receptor CD16 and the adhesion molecule L-selectin, three distinct neutrophil subsets, defined as CD16^DIM^ CD62L^BRIGHT^, CD16^BRIGHT^ CD62L^DIM^, or CD16^BRIGHT^ CD62L^BRIGHT^ have been identified in severely injured patients (118). CD16^BRIGHT^ CD62L^DIM^ neutrophils have been shown *in vitro* to potently suppress T-cell proliferation via a Mac-1-dependent mechanism that involves the release of hydrogen peroxide into the immunological synapse that forms between CD16^BRIGHT^ CD62L^DIM^ neutrophils and T cells (118). Importantly, CD16^BRIGHT^ CD62L^DIM^ neutrophils are a subset of mature neutrophils, thereby distinguishing them from the above-mentioned immature MDSCs who also suppress T-cell function (118). Currently, it is unknown as to whether suppressive CD16^BRIGHT^ CD62L^DIM^ neutrophils form part of the expanded peripheral neutrophil pool that is observed following TBI, although one study in patients with severe TBI recently reported a significant reduction in both CD62L surface density and the percentage of CD62L^+^ neutrophils, suggesting that suppressive neutrophils may be released into the circulation post-TBI (34). Hypothetically, their presence may be one explanation for the suppressed DTH skin test responses elicited by patients with severe TBI (25, 28, 61).

CD16^DIM^ neutrophils possess a “young” banded morphology, suggesting that these cells are immature granulocytic precursors (118). In a recent study of patients with septic shock, a subset of immature granulocytes (IGs) phenotypically defined as CD16^DIM^ 14^−^ 24^+^ were described and shown *in vitro* to elicit potent cytotoxicity against CD3^+^ lymphocytes (126). Interestingly, in these patients, a marked excess of CD16^DIM^ IGs was found to be significantly associated with both CD3^+^ and CD4^+^ T-cell lymphopenia (126). On this note, a feature of the acute immune response to severe TBI is a significant increase in circulating IG numbers (33), whose elevation precedes the well-described CD3 and CD4 T-cell lymphopenia that occurs in the hours and days following TBI (31, 40, 41). *Thus, could the emergence of CD16^*DIM*^*14*^−^*24**^+^*IGs into the circulation underlie the TBI-induced reduction in peripheral T cell numbers?* Although intriguing, proposing CD16^DIM^ 14^−^ 24^+^ IG-induced T-cell apoptosis as a mechanism of T-cell lymphopenia post-TBI is premature given that it has yet to be established whether CD16^DIM^ IGs cause T-cell death *in vivo* and the fact that no study to date has examined the surface phenotype of IGs released in response to TBI to ascertain whether this CD16^DIM^ 14^−^ 24^+^ subset is present. That said should future work address these issues and prove in the setting of TBI that such a relationship exists then a potential therapeutic target could emerge. Accelerating the maturation of immature CD16^DIM^ 14^−^ 24^+^ IGs through the systemic administration of agents capable of driving cellular differentiation could be one potential therapeutic avenue.

### Exposure to Damage-Associated Molecular Patterns – A Non-Neurological Explanation for TBI-Induced Innate Immune Dysfunction

Traumatic brain injury results in the emergence into the circulation (127) and cerebrospinal fluid (128) of mitochondrial DAMPs (mtDAMPs), a heterogeneous collection of mitochondrial-derived proteins and DNA that through binding to surface-expressed and endosomal residing pattern recognition receptors exhibit immune modulatory properties. In the case of isolated TBI, mtDAMPs originate from damaged neural tissue and through promoting the priming and activation of microglia and astrocytes are likely to orchestrate in part the inflammation-induced secondary phase of brain injury (129). Diffusion across a disrupted blood–brain barrier is the most obvious mechanistic explanation for how neural-derived mtDAMPs gain entry into the circulation post-TBI (127). However, results of a recent study suggest that this may not be the only mechanism. In a seminal publication, Plog et al. have shown that inhibition of the glymphatic system, a highly organized system of CSF-interstitial fluid exchange, prevents the delivery of endogenously produced markers of brain injury into the bloodstream (130). To date, S100B, GFAP, and neuron-specific enolase have all been shown in a murine model of TBI to exit the CNS and enter the circulation through this system (130). As mtDAMPs would be released from damaged neural tissue alongside these molecules then it is not inconceivable to think that the mtDNA that has been detected in the circulation of human TBI patients (127) gained entry into the periphery via the glymphatic system.

*In vitro*, we have shown mtDAMPs to be potent activators of resting human neutrophils, triggering a number of robust functional responses, which include ROS generation, degranulation, and IL-8 secretion (131), while *in vivo*, injection of mtDAMPs into mice results in among other things “priming” of circulating neutrophils (132). Therefore, given their presence in the circulation of TBI patients (127), exposure to mtDAMPs may be one mechanism behind the increased basal ROS generation that has been reported in the immediate aftermath of TBI (35).

From results derived from a series of *in vivo*, *in vitro*, and *ex vivo* studies, it has recently been suggested that prior exposure to mtDAMPs, in particular N-formyl peptides, may suppress neutrophil antimicrobial function upon secondary stimulation (119). Li et al. showed *in vitro* that pretreatment with fMLP significantly reduced neutrophil chemotaxis to leukotriene B4, while *ex vivo* studies revealed neutrophils isolated from trauma patients, in whom circulating levels of mtDAMPs are increased (132), exhibited suppressed chemotaxis to a range of chemoattractants as well as impaired neutrophil extracellular trap generation following PMA stimulation (119). Based on these observations, the phrase “DAMP-induced tolerance” was coined, which proposes that mtDAMPs may tolerize circulating neutrophils to subsequent pathogenic danger signals (119). Relating these findings to TBI, neutrophils isolated from patients with severe TBI have been shown *ex vivo* to exhibit significantly reduced ROS generation and phagocytosis when challenged with *E. coli* (36). *Thus, could it be that by initiating neutrophil activation in vivo, circulating mtDAMPs are responsible in part for the reduced anti-microbial activity of neutrophils post-TBI and hence the increased susceptibility of these patients to nosocomial infection?* To answer this question, a greater understanding of the type and amount of mtDAMPs released following TBI and the effect that prior exposure to mtDAMPs has on other antimicrobial functions of neutrophils besides chemotaxis and NET generation is needed. Interestingly, in the field of burns research, a recent study that enrolled patients with combined inhalation and burn injury reported that elevated levels of two DAMPs, namely hyaluronic acid and double-stranded DNA, in bronchial washings were significantly associated with bacterial respiratory infection during the first 14 days after injury (133). Although neutrophil function was not investigated directly in the study, alterations in neutrophil biology were proposed as one explanation for the relationship between DAMPs and the increased susceptibility of burns’ patients to infection, with the authors suggesting that DAMPs may trigger polarization of neutrophils toward an IL-10^+^ IL-12^−^ anti-inflammatory phenotype (133).

Defined as “the severely reduced capacity of a cell to respond to LPS during a second exposure to this stimulus” (134), endotoxin tolerance (ET) describes a transient refractory state, which in monocytes is characterized in part by reduced proinflammatory cytokine production and high surface expression of CD163, two features reminiscent of anti-inflammatory “M2” macrophages (135). Interestingly, mtDAMPs, in particular mtDNA, are potent inducers of ET in human monocytes (136). Fernández-Ruiz et al. (136) found that when challenged with LPS, monocytes pretreated with mtDNA exhibited among other things significantly reduced proinflammatory cytokine production and enhanced IL-10 secretion, leading to the suggestion that DAMPs may play a pivotal role in the refractory state of monocytes in “sterile” pathologies (136). On this note, significantly greater numbers of circulating anti-inflammatory “M2” monocytes and increased intracellular expression of IL-10 in monocytes has been reported post-TBI (30, 37), suggesting the presence of an immune-suppressive monocyte pool. If, as reported in other patient cohorts (136), future studies were to show that the elevation in circulating mtDNA that occurs following TBI (127) is associated with the development of monocyte ET and an increased incidence of infection, then an early assessment of the functional response of circulating monocytes to LPS may identify TBI patients at risk of developing nosocomial infection. In support of this concept, Pena et al. recently demonstrated in a cohort of critically ill patients that at the time of clinical presentation, a unique ET gene expression profile in PBMCs could be used to predict the development of sepsis and organ failure (134).

As described earlier, autoantibodies specific for CNS-residing proteins have been detected in the serum of patients that have suffered either mild (62) or severe (63) TBI. Given that damage to neural tissue results in the emergence into the circulation of mtDAMPs, *could these components lead to immune dysregulation in the TBI patient by triggering an autoimmune response?* Currently, no study to our knowledge has investigated this possibility. However, autoantibodies directed against mitochondria and mtDNA have been detected in patients with inflammatory disease (137) and neurodevelopmental disorders (138), and anti-mtDNA antibodies have been shown *in vitro* to drive a robust proinflammatory response from circulating immune cells (137). If replicated in the setting of TBI, one could envisage how after crossing the “leaky” BBB, autoantibodies against CNS-residing mtDAMPs could trigger an inflammatory reaction from local microglia and astrocytes that contributes to the onset of secondary brain injury.

In animal models of experimental brain ischemia, the nuclear-derived DAMP high mobility group box 1 (HMGB1) has been linked to the development of CNS injury-induced immune suppression (139). Released into the extracellular environment as a consequence of cellular necrosis or active secretion by immune cells, HMGB1 was shown *in vivo* to promote the expansion of MDSCs as well as partially mediate stroke-induced lymphocytopenia and monocyte hyporesponsiveness (139). Given that akin to stroke, TBI results in elevated plasma levels of HMGB-1 (140), *could the presence of this DAMP in the circulation underlie not only the well-established features of TBI-induced immune suppression (e.g., lymphocytopenia and immune exhaustion) but also those immune changes we have proposed may occur following TBI (e.g., emergence of MDSCs)?* A future study that measures immune function alongside HMGB-1 levels would address this question, and if a role for HMGB-1 in TBI-induced immune suppression was found then a therapeutic target for the prevention of immune dysfunction post-TBI could emerge.

## TBI and Peripheral Immune Suppression – Unanswered Questions and Future Opportunities

Despite the recent surge of interest in TBI and its impact on peripheral immunity (31–38, 141–143), our understanding of this subject remains equivocal at best. Thus, in this section, in an effort to move this field of research forward, a series of questions are posed, the answers to which we feel would provide much needed information not only on the mechanisms underlying TBI-induced changes in immunity but whether therapeutic intervention aimed at manipulating peripheral immune function has potential as a future treatment option for the hospitalized TBI patient.

### Q1. What are the Acute and Long-Term Effects of TBI on Peripheral Immune Function?

A question that has not been properly addressed in the field of TBI and trauma research in general is *how quickly following a traumatic insult do changes in peripheral immunity occur?* In the TBI studies described in this review, collection of the first blood sample postinjury has ranged from ≤3 to 72 h, a broad time-frame that may explain in part some of the conflicting observations that have been reported to date in respect of the impact of TBI on peripheral immunity. Without a complete understanding of the immediate immune response to TBI, it is likely that information that may be useful for the clinician when deciding upon treatment protocols for the injured patient is being missed. For instance, Rovlias et al. demonstrated the benefit of early blood sampling by showing that white blood cell counts in admission blood samples (within 2 h of injury) could independently predict patient outcome at 6 months (144). In another study, Pagowska-Klimek et al. measured neutrophil function in blood samples taken 0.5–2 h (mean 1 h) following isolated head injury in children and reported significantly reduced ROS generation following PMA and fMLP stimulation (145), demonstrating the immediate impact that TBI can have on peripheral immunity. However, whether this early impairment in neutrophil function resulted in an increased incidence of infection or poor outcome was not determined. Performing similar studies in adult patients that not only analyze the very early functional immune response to TBI but collect detailed information on a series of clinical outcomes, such as infection, development of sepsis, and mortality, would generate novel data that could have potential clinical benefit.

In addition to a lack of understanding of the immediate immune response to TBI, very little is known in respect of the long-term impact that TBI has on immune function. In the field of burns research, it has been reported that it takes a total of 3.5 months following thermal injury for the oxidative burst capacity of circulating neutrophils to return to a level that resembles that of healthy controls (146). In adult TBI patients, a significant reduction in the phagocytic activity of circulating neutrophils has been reported up to 2 weeks postinjury (35), while the immediate suppression in the frequency and absolute number of CD3^−^56^+^ NK cells that accompanies TBI is maintained up to 21 days postinjury (38). In rodent models of TBI, significantly reduced numbers of thymocytes, monocytes, and tissue macrophages as well as a decline in thymic mass has been recorded at 60 days postinjury (37). As these results suggest that TBI may have long-lasting detrimental effects upon the immune system, it would be interesting for future studies to assess, via a longitudinal approach, the extent to which TBI impacts upon the long-term functioning of the immune system especially since mortality as a result of infection has been reported in patients with severe TBI as late as 3 months postinjury (147).

### Q2. Could a Heterogeneous Pool of Circulating Neutrophils Explain the Alterations in Neutrophil Function Observed Post-TBI?

Neutrophilia is a well-described feature of the immune response to TBI (34, 35, 39), yet no study to date has examined the composition of this enlarged neutrophil pool. Although unknown in respect to TBI, severe traumatic injury results in a heterogeneous neutrophil pool that broadly consists of two distinct subsets, namely CD16^BRIGHT^ and CD16^DIM^ (118). Compared to their CD16^BRIGHT^ counterparts, CD16^DIM^ neutrophils exhibit significantly reduced ROS generation, phagocytosis, and bacterial killing (117, 148). Given that these three neutrophil functions are significantly impaired following severe TBI (35, 36, 40, 149)*, could it be that akin to other forms of traumatic injury, TBI results in a heterogeneous neutrophil pool?* Since all studies that have examined the impact of TBI on neutrophil biology to date have considered the circulating neutrophil pool to be a single homogenous population, the answer to this question is currently unknown. By addressing this issue, a greater understanding of the mechanisms that underlie TBI-induced immune suppression would be gained and potential therapeutic targets identified.

### Q3. Can Peripheral Immune Suppression and/or mtDAMPs Serve as a Biomarkers of Outcome in the Traumatically Brain Injured Patient?

Recently, a number of studies have demonstrated that changes in peripheral immunity are associated with and/or can predict outcome in TBI patients. Focusing primarily upon the inflammatory response to TBI, studies have shown increased levels of IL-6, IL-8, and IL-10 are associated with fatal outcome following severe TBI, with elevated IL-10 levels being associated with hospital mortality independent of patient age, GCS score, pupil reactivity at admission, and associated trauma (141, 142). In addition to mortality, raised serum IL-6 levels within 17 h of injury have been found to be a good prognostic marker for the development of raised intracranial pressure following isolated TBI (150), while an elevated IL-6:IL-10 ratio was associated with an increased risk of an unfavorable GOS score at 6 months postsevere TBI (143). Besides inflammatory cytokines, leukocytosis has been shown to be of prognostic significance in TBI patients. High white blood cell counts within the first day following injury have been found to be associated with a longer length of hospital stay (151), mortality (33), and unfavorable neurological outcome at 6 months, with this latter association independent of GCS score, pupillary reaction, age, and intracranial diagnosis (144, 151).

Whether the peripheral immune suppression that occurs post-TBI can be used to predict the development of infection is currently unknown, although this approach has proven to be successful in other cohorts of patients with CNS injury. In stroke patients, in whom infections are associated with higher mortality rates and poor functional outcome (152), several features of stroke-induced immune depression have been shown to be associated with and/or independently predict the development of nosocomial infection. These include: (1) reduced monocytic HLA-DR expression and low CD4^+^ T-cell counts (153, 154), (2) reduced *ex vivo* TNF-α release by monocytes (155), and (3) elevated plasma IL-10 levels (156). In addition, it has recently been postulated that a rise in the frequency of circulating CD64^+^ neutrophils 1 week after stroke may serve as a marker by which to identify an inflammatory response developing as a consequence of infection (157). In the context of TBI-induced immune suppression, no study to our knowledge has examined whether alterations in neutrophil surface phenotype can predict the development of infection, a reflection of the paucity of data in this area of research. There have however been reports of reduced ROS production by neutrophils isolated from infected TBI patients compared to non-infected patients (36). That said this relationship was not predictive and the potential impact of confounders were not considered. This lack of comprehensive statistical analysis is also a limitation of a study where infected patients with severe TBI were found to have significantly lower circulating frequencies of CD3^+^56^−^, CD3^+^4^+^ and CD3^+^8^+^ T cells as well as CD3^−^56^+^ NK cells at the time of infection occurrence when compared to non-infected patients (31). Thus, although associated with nosocomial infection, it remains to be established whether TBI-induced immune suppression can be used to independently predict the development of infection. Given the range of adverse outcomes that are associated with infection in TBI patients (11, 14, 15, 17, 20, 21) and the benefit that early detection may have on patient health, addressing this issue should form a significant part of future TBI research.

Aside from immune function, *could the presence of mtDAMPs serve as a biomarker of injury severity or patient outcome post-TBI?* In other fields of trauma research, plasma levels of mtDNA have been found to (i) positively correlate with injury severity score (ISS) (158–160), (ii) be significantly higher in admission samples from non-survivors compared to survivors (158, 161, 162), and (iii) be predictive of and/or associated with the development of a systemic inflammatory response or multiple organ failure (160, 161). However, whether mtDAMPs are suitable diagnostic or prognostic markers in TBI is currently unclear. In the only study to our knowledge that has measured circulating mtDAMPs in human TBI patients, Wang et al. found that although significantly increased when compared to healthy controls, plasma mtDNA levels on admission did not correlate with either GCS score or ISS (127). Similarly, no association was observed between mtDNA levels in admission CSF samples and GCS score in a cohort of pediatric TBI patients (128). However, CSF mtDNA concentration was found to predict with a reasonable degree of specificity (84.2%) patient outcome at 6 months (128). More recently, in a porcine model of focal and diffuse TBI, Kilbaugh et al. reported a significant increase in relative mtDNA copy numbers in peripheral blood at 6 and 25 h postinjury (163). Interestingly, this increase was shown to correlate with a reduction in cerebral mitochondrial respiration, leading to the proposal that enumeration of relative mtDNA copy numbers in peripheral blood could represent a novel signature of cerebral mitochondrial dysfunction post-TBI (163).

### Q4. Is Reversing TBI-Induced Immune Suppression a Potential Therapeutic Strategy for the Treatment of Nosocomial Infections in the Hospitalized TBI Patient?

The majority of studies that have investigated whether the immune system is a potential therapeutic target for the treatment of TBI have focused upon developing immune suppressive strategies as a means of treating and/or preventing the secondary injury phase of TBI. To date, these strategies have included (1) neutrophil depletion, which reduced edema formation and attenuated brain tissue loss (164), (2) ghrelin therapy, which reduced peripheral monocyte and neutrophil recruitment to the site of brain injury (165), and (3) blocking leukotriene synthesis, which resulted in an attenuation in brain swelling and reduced blood–brain barrier permeability (166). Given the interest in preventing secondary injury following TBI, very little is known, particularly in humans, as to whether counteracting TBI-induced immune suppression may be a useful approach for the prevention of infection in the hospitalized TBI patient. While it may seem counterintuitive to enhance immune activity to eradicate infection given that immune hyperactivity is central to the pathophysiology of secondary brain injury, it has been suggested that such approaches would only commence when any secondary injuries are controllable (147).

In a randomized, placebo-controlled, double-blind, multicentre phase II study, Heard et al. prospectively investigated whether the use of prophylactic recombinant human granulocyte colony-stimulating factor (G-CSF) could reduce the frequency of nosocomial infections in patients with acute TBI (167). They found that while G-CSF treatment had no impact on the frequency of pneumonia or urinary tract infections, it was associated with a significant reduction in the frequency of primary bacteremias (167). However, the study found no beneficial effect for G-CSF therapy on patient mortality or length of hospital stay (167). Following on from this work, Ishikawa et al. investigated whether G-CSF treatment could reduce the incidence of life-threatening infections in eight patients with severe TBI that were undergoing combined therapy with barbiturates and mild hypothermia, a treatment protocol known to increase infection risk (147). The group found that compared to patients who did not receive G-CSF therapy, prevalence rates of pneumonia and meningitis were significantly lower in G-CSF treated patients (147). Moreover, compared to baseline values, G-CSF treatment resulted in a significant increase in circulating neutrophil numbers and antimicrobial function with respect to phagocytic and bactericidal activities, suggesting that an enhancement in immune function had protected the patients from contracting nosocomial infection (147). Given the potential clinical significance of this study, it is surprising that in the 16 years since its publication no group has taken this research forward, especially since during this time period, immunomodulation in other forms of traumatic injury has proven successful in protecting against the development of post-traumatic infection (168, 169). Thus, it remains to be established whether therapies aimed at restoring immune function would be of benefit to the hospitalized TBI patient in terms of reducing their susceptibility to infection.

## Conclusion

In this review article, we have proposed that the emergence into the circulation of hematopoietic cells with suppressive activity underlies in part the immune suppression that is observed following TBI. If future studies prove this hypothesis to be correct then *could these cells be manipulated for the clinical benefit of the hospitalized TBI patient in respect of reducing infection risk?* Interestingly, therapeutic interventions are being trialed in other cohorts of patients to address similar questions. For instance, a series of strategies aimed at manipulating the function of MDSCs are currently under clinical investigation as a novel treatment for cancer (125), while in the field of sepsis research, groups are examining whether it is possible to remove individual neutrophil subsets from the circulation. On this note, *ex vivo* filtration of blood from patients with septic shock has been found to preferentially remove neutrophils with an activated phenotype (CD11b^high^, CD64^high^, and CXCR1/2^low^) (170). If, as we have proposed, suppressive CD16^BRIGHT^ CD62L^DIM^ neutrophils emerge into the circulation following TBI and are involved in TBI-induced immune suppression then it would be of interest to determine whether a similar filtration approach to remove this specific neutrophil subset could be beneficial in terms of reducing infection risk in TBI patients.

## Conflict of Interest Statement

The authors declare that the research was conducted in the absence of any commercial or financial relationships that could be construed as a potential conflict of interest.
